# Influences of Community Interventions on Zika Prevention Behaviors of Pregnant Women, Puerto Rico, July 2016–June 2017^1^

**DOI:** 10.3201/eid2412.181056

**Published:** 2018-12

**Authors:** Giulia Earle-Richardson, Christine Prue, Khadija Turay, Dana Thomas

**Affiliations:** Centers for Disease Control and Prevention, Atlanta, Georgia, USA

**Keywords:** Zika virus, health behavior, program effectiveness, maternal health, mosquito bed nets, interpersonal communication, risk perception, viruses, pregnancy, Puerto Rico

## Abstract

We assessed how community education efforts influenced pregnant women’s Zika prevention behaviors during the 2016 Centers for Disease Control and Prevention–Puerto Rico Department of Health Zika virus response. Efforts included Zika virus training, distribution of Zika prevention kits, a mass media campaign, and free home mosquito spraying. We used telephone interview data from pregnant women participating in Puerto Rico’s Women, Infants, and Children Program to test associations between program participation and Zika prevention behaviors. Behavior percentages ranged from 4% (wearing long-sleeved shirt) to 90% (removing standing water). Appropriate mosquito repellent use (28%) and condom use (44%) were common. Receiving a Zika prevention kit was significantly associated with larvicide application (odds ratio [OR] 8.0) and bed net use (OR 3.1), suggesting the kit's importance for lesser-known behaviors. Offer of free residential spraying was associated with spraying home for mosquitoes (OR 13.1), indicating that women supported home spraying when barriers were removed.

In early 2016, in response to the rising number of Zika virus infections in Puerto Rico and the devastating effects of Zika infection during pregnancy (*1*), the Puerto Rico Department of Health (PRDOH) activated its emergency operations center, with support from the US Centers for Disease Control and Prevention (CDC) (*2*). Because there is currently no Zika virus vaccine and no known measures can prevent prenatal mother-to-child transmission (*3*), personal protection measures and home vector control are the only feasible protections for most pregnant women. To maximize these self-protection behaviors, the response introduced 4 different community Zika prevention behavior promotion interventions. Health behavior interventions can change behavior by addressing behavioral barriers, by creating or enhancing incentives, and by increasing persons’ capabilities and opportunities to perform the behavior (*4*).

## Interventions

### PRDOH Women, Infants, and Children Program Zika Orientation

During the tracking period, all newly enrolled pregnant women at 1 of the island’s 92 Women, Infants, and Children (WIC) clinics were given a 20–30-minute presentation on Zika virus infection and prevention. Orientation (individually or in small groups) was provided by the nutrition educator or, during the peak of the epidemic, by a Zika educator provided by CDC. The primary advantages of this counseling approach are interpersonal communication (including answering questions) and how easily it can be integrated into existing trusted programs, such as WIC prenatal visits (*5*,*6*).

### Zika Prevention Kit Distribution

The Zika prevention kit (ZPK) was a tote bag containing insect repellent, condoms, a mosquito bed net, larvicide, and printed Zika education materials. Approximately 26,000 ZPKs were distributed in Puerto Rico (CDC–Puerto Rico Department of Health, unpub. data, April 26, 2017). Whenever possible, the ZPK was given to the pregnant woman at the same time as the WIC Zika orientation. Prevention kits enable healthy behavior by putting needed items in persons’ hands but also by providing a visual reminder of the recommended behavior. Similar home infection prevention kits were used during the Zika response in the US Virgin Islands (*7*) and during the Ebola epidemic in West Africa (*8*–*10*) to provide home caregivers with tools to prevent virus transmission. Only HIV infection prevention kits have been evaluated to date; these preliminary evaluations indicate kit popularity and suggest supportive effects (*11*,*12*).

### Detén el Zika Campaign

The Detén el Zika (“This Is How We Stop Zika”) campaign disseminated strategically designed Zika prevention messages through television, radio, print, and social media channels (*13*). The television advertisement included a montage showing couples or pregnant women and their families performing the following behaviors: using repellent, using condoms, using bed nets, removing standing water, and installing screens. Mass media campaigns have the advantage of reaching multiple audiences (including partners, families, and pregnant women not enrolled in WIC) with repeating messages that appeal cognitively and emotionally by showing relatable images of women taking preventive steps and by showing a healthy baby (*14*).

### Offer of Free Residential Mosquito Spraying Services

When pregnant women attended their WIC appointments, they were also offered a free residential mosquito spraying service. Upon consent, WIC provided women’s contact information to a contracted professional spraying service. Across the island, ≈3,400 homes were sprayed through this program. For this analysis, this intervention is defined as the offer of free residential spraying services, meaning that women who report being offered the free service are classified as exposed to the intervention, regardless of whether they chose to use the service. In this way, we can determine whether having free residential spraying services available affected the overall frequency of spraying the home (or yard) for mosquitoes.

Although we might intuit that making residential spraying free would increase use, the literature contains inconsistent evidence about whether removing cost barriers increases vector control behavior (*15*–*17*). This offer of free residential mosquito spraying was discontinued in August 2016 after a CDC evaluation found that mosquito populations in and around sprayed homes had not changed, probably as a result of movement of mosquitoes from nearby homes (*18*).

## Intervention Implementation Monitoring

As these interventions were being implemented, the response behavioral science team conducted monthly telephone interviews of a random sample of 300 pregnant women participating in WIC to provide feedback to the response leadership about intervention exposure and women’s Zika prevention behavior. A subset of 150 respondents were asked about their performance of the following 10 CDC-recommended behaviors: using mosquito repellent, using condoms, abstaining from sex, wearing long-sleeved shirts, wearing long pants, sleeping under a bed net, removing or covering standing water, applying larvicide (in water that cannot be removed), putting screens on windows and doors, and spraying home and yard for mosquitoes. This assessment continued until June 2017, when PRDOH declared the Zika epidemic over (*19*). During 2016–2017, a total of 9 monthly (in 2017, bimonthly) interview rounds were conducted. Our analysis addresses the following: 1) the proportion of pregnant respondents reached by the 4 interventions and the factors associated with exposure; 2) the Zika prevention behaviors that were most widely practiced and that were most strongly associated with exposure to interventions; and 3) additional factors associated with Zika prevention behavior that might provide insight into how the interventions influenced behavior.

## Methods

### Interview Population and Sampling

Each month during July–December 2016 and every 2 months during February–June 2017, a random sample of 950 pregnant women >18 years of age (317 women per pregnancy trimester) was drawn from the WIC enrollment database of 10,000–12,000 women currently enrolled (and not previously contacted) for interviews. Vital statistics data indicate that 87% of women giving birth in Puerto Rico in 2016 were enrolled in WIC (Table 1). The calling list was divided among interviewers so that some began with first trimester women, some with second, and some with third. As part of the Zika response, these interviews were determined to be nonresearch public health practice and were approved by the US Office of Management and Budget (control no. 0920–1196). Before asking women for their verbal agreement to participate, interviewers explained the purpose of the data collection, the fact that their participation and all responses would be kept confidential, and that they could discontinue the interview any time without any penalty. The 3 groups of callers continued until 300 total interviews were completed. The interview had 2 parts, administered 2 weeks apart. Those women who consented to complete part 2 were called in the same order as for part 1 until 150 interviews were completed.

**Table 1:** Demographic characteristics of all women giving birth in 2016 and interview participants, Puerto Rico, July 2016–June 2017*

Characteristic	Sample size, no. (%)	Women who gave birth in 2016, no. (%)†	
		>18 y of age	All ages
Total sample	1,329 (100)	27,230 (100)	28,257 (100)
Age group, y			
<18‡	0	0	1,027 (4)
18–22	353 (27)	7,963 (29)	7,963 (28)
23–25	324 (24)	5,436 (20)	5,436 (19)
26–29	319 (24)	5,884 (22)	5,884 (21)
>30	333 (25)	7,947 (29)	7,947 (28)
Total sample	1,329 (100)	27,230 (100)	28,257 (100)
Educational attainment			
Some high school or less	24 (3)	427 (2)	579 (2)
Attended or completed 12th grade	285 (31)	9,105 (34)§	9,958§ (35)
Attended or completed university	545 (60)	15,648 (58)	15,670 (55)
Attended or completed graduate program	55 (6)	2031 (8)	2,031 (7)
Total sample	909¶ (100)	27,230 (100)	28,257 (100)
Participation in WIC program#	1,329 (100)	23,679 (87)	24,671 (87)
Geographic region of Puerto Rico			
Metropolitan San Juan	203 (15)	2,864 (11)	2,955 (10)
Metropolitan Bayamon	182 (14)	1,556 (6)	1,597 (6)
Nonmetropolitan regions	941 (71)	22,810 (83)	23,705 (84)
Total sample	1,327 (100)	27,230 (100)	28,257 (100)

*NCHS, National Center for Health Statistics (Centers for Disease Control and Prevention); WIC, Women, Infants, and Children Program (US Department of Agriculture Food and Nutrition Service). †Source: NCHS’s US Territories, 2016 natality public use file (https://www.cdc.gov/nchs/data_access/vitalstatsonline.htm). ‡Because women had to be >18 years of age to participate, the <18 age category is empty for the WIC sample. §In the NCHS data, this group includes 9th–12th grade, not just 12th grade. ¶The educational attainment data in the WIC dataset (n = 909) were incomplete. The data here represent 68% of the total sample of 1,329. #Source: WIC (https://www.fns.usda.gov/wic/women-infants-and-children-wic).

### Data Collection

The interview consisted of questions about Zika knowledge, attitudes, sources of information, exposure to prevention interventions, and Zika prevention behaviors. Many of the questions involved binary (e.g., yes or no) or scaled (e.g., never, rarely, sometimes, frequently, or often) responses. Others were questions in which the interviewer did not provide response options to the participant but coded the response according to a checklist. Although Zika infection status was not an interview question, if a participant disclosed that she was Zika positive, the interview was excluded from the dataset. This exclusion was made because Zika virus infection confers immunity and therefore an already positive woman would have no reason to take prevention steps.

### Definition of Intervention Exposure

Respondents were asked if they had received the WIC Zika orientation, the ZPK, or the offer of free home spraying. They were also asked if they had seen communications from the Detén el Zika campaign. Any woman answering affirmatively to any of these questions was defined as exposed to the corresponding intervention.

### Data Analysis

#### Calculation of Zika Prevention Behavior Variables

Because the original interview instrument included multiple questions about each Zika prevention behavior without any clear formula for integrating question responses into a single variable (1 per behavior), analysts had to create such a formula. For example, some questions asked whether a woman performed the behavior any time during pregnancy (or during the previous day or week) (yes or no), whereas others used ordinal frequency scales (e.g., never, sometimes, or always). In addition, a Zika prevention behavior could be reported in response to the question, “What actions have you taken to protect yourself from being infected by the Zika virus?”

To describe women’s Zika prevention behavior as completely as possible, analysts created behavior variables that incorporated 2, 3, or more questions. We prioritized time-bound, behavior-specific questions, such as, “How often did you use mosquito repellent in the past week?” (never, sometimes, or always), over a more general question such as, “What actions have you taken to protect yourself from being infected with the Zika virus?” Among the behavior-specific questions, those questions with multilevel response options were prioritized over yes or no or dichotomous response questions, given that the greater number of response options yielded more information. Zika prevention behavior variables were then created with ordinal scales, combining the most detailed behavior-specific question available for the behavior with other questions that might serve to increase the number of levels of Zika prevention behavior. Once preliminary scales were created, frequencies and plots were reviewed by behavioral scientists and epidemiologists involved with the Zika response to achieve a consensus on the final composition. We have compiled a list of all candidate questions and final variables (Technical Appendix).

#### Statistical Methods

Analysts calculated frequencies of intervention exposure by interview month and demographic characteristics. In addition, because the interventions sought to increase Zika prevention behavior by increasing a woman’s concern about Zika, her confidence in her ability to protect herself, and involvement of partners and families in Zika prevention, variables representing these constructs were tested for associations with intervention exposure and Zika prevention behaviors. All analyses were conducted with SPSS 21.0 (IBM Corp., Armonk, NY, USA).

Analysists used logistic regression modeling to estimate odds ratios (ORs) for the likelihood of performing recommended Zika prevention behaviors by exposure to 1 of the Zika prevention interventions while controlling for the effects of age, education, pregnancy trimester, poverty, calendar month of interview, and exposure to other interventions. For these models, Zika prevention behavior variable responses were collapsed into dichotomous (yes or no) variables, indicating whether a respondent had performed the ideal behavior (e.g., always uses a condom) or not. In the case of mosquito repellent use, the 2 top levels, which both include the response always, were combined to make the top level.

Because the WIC orientation reached nearly all respondents, the naturally occurring control group of unexposed women was very small, causing concerns about small cell size in models with many covariates (*20*). Conversely, a small exposure group was a concern with the offer of free residential mosquito spraying. Therefore, these 2 interventions were modeled separately from ZPK distribution and Detén el Zika, which were modeled together. In addition, sparsity concerns led us to consolidate the calendar month of interview variable into 1 representing 3-month intervals.

## Results

### Participant Characteristics

Our sample encompassed 1,329 pregnant WIC participants interviewed during July 2016–June 2017 (Table 1). Among eligible women (i.e., >18 years of age, pregnant, and not Zika positive), the response rate was 79%. Age and educational attainment distributions of the sample were similar to the general population of women giving birth in Puerto Rico in 2016 (*21*), whereas urban residence is somewhat higher.

### Women’s Exposure to 4 Zika Prevention Interventions

Women reported exposure to the 4 interventions as follows: WIC Zika orientation (93%), ZPK distribution (75%), Detén el Zika campaign (51%), and offer of free residential mosquito spraying (68% for the months it was running and 34% over the entire period). Pregnancy trimester was statistically significant for association with exposure to all 4 interventions, whereas calendar month of interview was significantly associated with 3 interventions (Table 2). No significant associations were observed in terms of age, education, poverty, or rurality.

**Table 2:** Respondents exposure to 4 Zika prevention interventions, by demographic characteristics and calendar month, Puerto Rico, July 2016–June 2017*

Characteristic	Sample	Received WIC Zika orientation			Received ZPK			Exposed to Detén el Zika campaign			Offered free home spraying	
		No	Yes		No	Yes		No	Yes		No	Yes
Pregnancy trimester at interview												
1st	26.8	8.4	91.6		32.9	67.1		52.2	47.8		68.1	31.9
2nd	48.6	8.2	91.8		24.6	75.4		45.9	54.1		71.7	28.3
3rd	24.6	3.7	96.3		16.9	83.1		53.4	46.6		52.8	47.2
Total no.	1,329	95	1,230		324	976		600	616		873	448
p value		**0.019**			**0.000**			**0.052**			**0.000**	
Calendar month of interview												
Jul 2016	11.2	4.8	95.2		4.8	95.2		62.9	37.1		29.7	70.3
Aug 2016	11.1	8.2	91.8		23.8	76.2		59.2	40.8		29.3	70.7
Sep 2016	10.1	6.0	94.0		31.3	68.7		44.4	55.6		34.6	65.4
Oct 2016	11.3	10.7	89.3		41.3	58.7		31.6	68.4		65.8	34.2
Nov 2016	11.3	8.0	92.0		31.3	68.7		35.0	65.0		70.1	29.9
Dec 2016	11.3	4.0	96.0		30.0	70.0		36.1	63.9		72.7	27.3
Feb 2017	11.3	6.7	93.3		20.7	79.3		55.2	44.8		97.3	2.7
Apr 2017	11.3	5.3	94.7		16.0	84.0		68.1	31.9		94.7	5.3
Jun 2017	11.2	10.7	89.3		22.1	77.9		52.9	47.1		96.6	3.5
Total no.	1,329	203	1,230		324	976		600	616		873	418
p value		0.225			**0.000**			**0.000**			**0.000**	
Age group, y												
18–22	26.6	6.8	93.2		22.3	77.7		51.0	49.0		66.2	33.8
23–25	24.4	7.4	92.6		23.5	76.5		52.1	47.9		68.5	68.5
26–29	24.0	6.9	93.1		27.0	73.0		44.1	55.9		64.9	35.1
>30	25.1	7.5	92.5		27.2	72.8		49.8	50.2		64.8	35.2
Total no.	1,329	95	1,230		324	976		600	616		873	448
p value		0.981			0.356			0.217			0.723	
Educational attainment												
Some high school or less	2.6	0.0	100.0		21.7	78.3		54.5	45.5		66.7	33.3
Attended or completed 12th grade	31.4	7.4	92.6		22.3	77.7		49.4	50.6		66.1	33.9
Attended or completed university	60.0	6.1	93.9		23.6	76.4		49.9	50.1		62.2	37.8
Attended or completed graduate program	6.1	7.3	92.7		25.6	74.1		36.2	63.8		58.2	41.8
Total no.	909	58	848		207	681		404	419		572	332
p value		0.512			0.934			0.315			0.579	
Population in poverty in Zip code, % quartiles†												
>55 below poverty	25.0	5.1	94.9		22.8	77.2		49.5	50.5		65.2	34.8
49–54 below poverty	25.3	7.9	92.1		25.6	74.4		43.7	56.3		67.5	32.5
43–48 below poverty	25.1	6.7	93.3		23.1	76.9		51.6	48.4		64.2	35.8
<43 below poverty	24.5	8.8	91.2		29.2	70.8		53.1	46.9		68.1	31.9
Total no.	1,255	89	1,163		309	918		566	579		826	421
p value		0.305			0.234			0.125			0.700	
Municipality population												
>200,000	63.5	6.1	93.9		23.1	76.9		48.6	51.4		66.0	34.0
>100,00–200,000	9.9	10.6	89.4		31.3	68.7		50.4	49.6		61.8	38.2
>50,000–100,000	12.6	7.7	92.3		27.9	72.1		46.8	53.2		63.3	36.7
<50,000	14.0	8.8	91.2		26.3	73.7		56.1	43.9		72.5	27.5
Total no.	1,326	91	1,184		313	937		578	589		839	431
p value		0.213			0.163			0.328			0.187	

*All data are percentages unless otherwise indicated. Statistically significant differences (p<0.05 by χ^2^ test) are shown in boldface. WIC, Women, Infants, and Children Program (US Department of Agriculture Food and Nutrition Service); ZPK, Zika prevention kit. †Source: US Census Bureau American Community Survey, 2016. American Community Survey 5-year estimates, Table S1701 (generated by G.B.E. using American Fact Finder, 2018 Feb 24).

**Figure 1:**
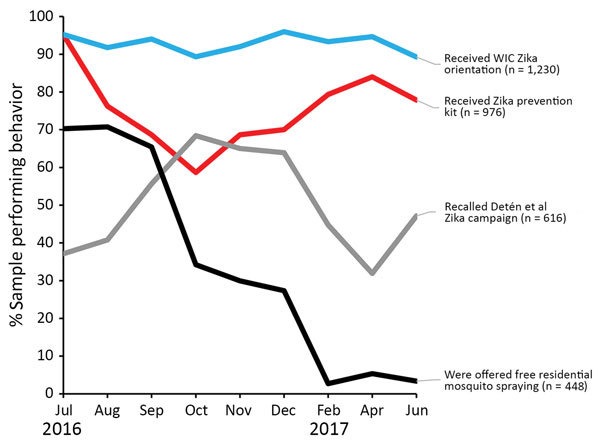
Percentage of pregnant women reporting exposure to 4 Zika prevention interventions, by interview month, Puerto Rico, 2016–2017. August 12, 2016: President declares Zika in Puerto Rico a “public health emergency” (https://www.reuters.com/article/us-health-zika-usa/u-s-declares-a-zika-public-health-emergency-in-puerto-rico-idUSKCN10N2KA). September 30, 2016: Free residential spraying discontinued. Women who report the offer through December are referring to receiving the offer before September. October 28, 2016: First baby born with microcephaly in Puerto Rico (https://www.nytimes.com/2016/10/29/health/zika-microcephaly-puerto-rico.html). June 5, 2017: Zika epidemic declared over by Puerto Rico Department of Health (https://www.businesswire.com/news/home/20170605006235/en/Puerto-Rico-Department-Health-Declared-2016-Zika). WIC, Women, Infants, and Children Program (US Department of Agriculture Food and Nutrition Service).

Graphed by calendar month of interview (Figure 1), exposure to the WIC Zika orientation remained consistently high (89%–96%). ZPK distribution began high (95%), dropped in October, then rebounded. Detén el Zika campaign exposure began much lower (37%), then steadily increased through October (68%), dropped off, and rose again in 2017. Exposure to the offer of free residential mosquito spraying started at 70% in July 2016, then dropped precipitously after September.

Intervention exposure was more often significantly associated with family or interpersonal communication variables than with individual risk variables (Table 3). The same pattern was observed for associations with Zika prevention behaviors (data not shown): “frequency of talking to family and friends about Zika” was significantly associated with 10 behaviors and “aware of Zika prevention actions of family” with 5 behaviors, whereas all 3 individual risk perception–related variables were associated with <3 behaviors.

**Table 3:** Associations between Zika prevention intervention exposure and interpersonal communications about Zika and personal risk perceptions, Puerto Rico, July 2016–June 2017*

Variable	Sample	Received WIC Zika orientation			Received ZPK			Exposed to Detén el Zika campaign			Offered free home spraying	
		No	Yes		No	Yes		No	Yes		No	Yes
Family and interpersonal communication												
Frequency of talking to family and friends about Zika												
Not at all	10.7	8.4	10.8		9.9	11.1		14.5	7.3		12.7	6.7
Only once or twice	16.2	21.1	15.9		16.7	16.1		17.7	15.3		17.4	13.6
Sometimes	32.7	45.3	31.8		34.9	32.3		33.0	32.5		33.3	31.5
Often	22.0	16.8	22.4		18.5	22.7		19.2	23.5		20.3	25.2
Every day	18.4	8.4	19.2		20.1	17.8		15.7	21.4		16.3	23.0
Total no.	1,329	79	1,230		600	616		324	976		873	448
p value		**0.009**			0.472			**0.000**			**0.000**	
Aware of Zika prevention actions of family												
No	38.3	38.8	38.2		38.2	38.4		46.0	31.0		41.4	30.9
Yes	61.7	61.2	61.8		61.8	61.6		54.0	69.0		58.6	69.1
Total no.	1,168	85	1,081		511	561		314	850		818	343
p value		0.910			0.966			**0.000**			**0.001**	
Individual risk perception												
How concerned women feel about Zika												
Not at all concerned	8.2	7.4	8.3		5.6	9.0		8.8	7.8		8.9	6.7
Slightly concerned	16.4	13.7	16.7		14.8	17.1		17.9	15.6		17.7	13.8
Somewhat concerned	21.1	20.0	21.2		20.7	21.1		21.7	21.1		21.6	20.3
Moderately concerned	27.3	33.7	26.6		30.2	26.4		27.4	26.8		27.5	26.8
Extremely concerned	27.0	25.3	27.3		28.7	26.4		24.2	28.7		24.3	32.4
Total no.	1,328	95	1,229		599	616		324	975		872	448
p value		0.665			0.182			0.435			**0.019**	
How likely women feel they will become infected with Zika												
Extremely unlikely	10.0	9.7	10.0		8.1	10.8		10.2	9.9		12.0	5.9
Unlikely	37.4	36.6	37.4		37.4	37.4		36.6	38.0		36.8	38.4
Neither likely nor unlikely	30.6	32.3	30.5		31.5	30.2		30.3	31.4		31.0	30.2
Likely	19.4	19.4	19.4		20.9	18.8		19.3	18.8		18.0	22.1
Extremely likely	2.7	2.2	2.7		2.2	2.8		3.6	2.0		2.1	3.4
Total no.	1,306	93	1,209		587	606		321	957		855	443
p value		0.994			0.607			0.549			**0.003**	
Confidence in ability to protect self and baby from Zika												
Not confident at all	1.1	2.1	1.1		1.9	0.9		1.2	1.3		1.3	0.9
Somewhat unconfident	9.9	16.0	9.4		10.3	9.6		10.1	9.3		10.0	9.5
Not confident or unconfident	22.3	27.7	21.8		20.6	22.3		24.7	20.2		21.1	24.8
Confident	49.5	45.7	49.9		48.9	50.1		49.5	49.8		50.7	47.1
Very confident	17.2	8.5	17.9		18.4	17.1		14.6	19.3		16.8	17.8
Total no.	1,319	94	1,221		596	610		321	969		867	444
p value		**0.030**			0.634			0.139			0.530	

*All data indicate percentages unless otherwise indicated. Statistically significant differences (p<0.05 by χ^2^ test) are shown in boldface. WIC, Women, Infants, and Children Program (US Department of Agriculture Food and Nutrition Service); ZPK, Zika prevention kit.

### Pregnant Women’s Zika Personal Protection Behaviors

Frequencies of recommended personal protection behaviors (i.e., the top level on the ordinal scale) ranged from 4% (wearing long-sleeved shirt) to 44% (condom use) (Table 4). Although just over half of women reported using repellent always, fewer (28%) reported the top category, “used always and reported reapplying it.” Among the interventions, exposure to the WIC Zika orientation showed the greatest exposed versus not exposed frequency differences for the top behavior levels (Tables 4, 5).

**Table 4:** Zika personal protection behaviors among pregnant women, by exposure to 4 interventions, Puerto Rico, July 2016–June 2017*

Behavior	Entire sample	Received WIC Zika orientation			Received ZPK			Exposed to Detén el Zika campaign			Offered free home spraying	
		Yes	No		Yes	No		Yes	No		Yes	No
Mosquito repellent use												
Always, reported reapplying	28.3	29.1	18.9		29.7	24.5		31.2	25		32.6	26.1
Always, did not report reapplying	23.9	23.5	28.4		24.1	23.5		27.6	21.5		23.9	23.9
Usually or most of the time	25.9	26.4	21.1		25.9	26		23.1	28.4		23.2	27.1
Sometimes	13.0	12.8	14.7		12.2	15.2		11.7	14.5		13.2	13.1
Rarely or seldom	4.6	4.4	7.4		4.7	4.0		3.9	4.7		4.0	4.9
Never	4.2	3.8	9.5		3.4	6.8		2.6	5.8		3.1	4.8
Total no.	1,328	1,229	95		976	323		614	597		448	873
p value		**0.018**			**0.016**			**<0.001**			**0.012**	
Condom use†												
Always	44.1	45.3	31.6		45.1	42.6		44.2	26.3		42.5	44.8
Sometimes	29.3	29.5	24.1		30.6	25.8		28.7	26.3		28.3	29.9
Never	26.6	25.2	44.3		24.3	31.6		27.2	47.4		29.2	25.3
Total no.	1,047	964	79		768	256		491	464		353	689
p value		**0.001**			0.130			**0.001**			0.266	
Bed net use												
Slept under bed net yesterday	14.8	15.4	7.4		17.7	6.8		16.1	13.8		13.8	15.3
Did not use yesterday, reports use generally	4.9	5.2	1.1		5.7	2.5		4.2	4.7		3.1	5.8
Did not use yesterday, does not report use generally	80.3	79.4	91.6		76.5	90.7		79.7	81.5		83	78.8
Total no.	1,329	1,230	95		976	324		616	600		448	873
p value		**0.005**			**<0.001**			0.390			0.094	
Wearing long pants												
Wearing now, every day, all day	21.3	21.4	21.1		20.6	23.5		21.2	20.8		20.6	21.5
Wearing now, every day, part of day	19.2	19.5	15.8		18.7	21.0		20.4	18.3		19.7	19.0
Wearing now, does not wear every day	20.0	20	21.1		20.0	19.4		20.5	20		17.7	21.3
Not wearing long pants now	39.4	39.1	42.1		40.7	36.1		40.8	37.9		41.9	38.1
Total no.	1,327	1,228	95		974	324		614	600		446	873
p value		0.549			0.098			0.378			0.402	
Sexual abstinence												
Had no sex during pregnancy	20.2	20.7	15.8		20.3	19.9		31.2	25.0		20.6	19.9
Had sex during pregnancy	79.8	79.3	84.2		79.7	80.1		80.6	78.2		79.4	80.1
Total no.	1,324	1,225	95		973	322		614	597		447	869
p value		0.256			0.855			0.303			0.773	
Wearing long-sleeved shirt												
Wearing now, every day, all day	3.9	3.8	5.3		3.7	4.7		77.7	79.3		4.0	3.8
Wearing now, every day, part of day	6.7	6.7	7.4		7.2	5.6		6.4	7.2		6.9	6.7
Wearing now, does not wear every day	10.6	10.8	7.4		9.9	13.7		11.1	10.4		8.9	11.5
Not wearing long sleeves now	78.7	78.6	79.8		79.3	79.3		79.3	83.5		80.1	78
Total no.	1,325	1,227	94		974	322		614	598		448	869
p value		0.915			0.289			0.464			0.457	

*All data indicate percentages unless otherwise indicated. Statistically significant differences (p<0.05 by Mann-Whitney U nonparametric test) are shown in bold. WIC, Women, Infants, and Children Program (US Department of Agriculture Food and Nutrition Service); ZPK, Zika prevention kit. †Among those reporting having had sex during pregnancy.

**Table 5:** Zika home protection behaviors among pregnant women, by exposure to 4 interventions, Puerto Rico, July 2016–June 2017*

Behavior	Samples, % (no.)	Received WIC Zika orientation			Received ZPK			Exposed to Detén el Zika campaign			Offered free home spraying	
		Yes	No		Yes	No		Yes	No		Yes	No
Removing (or covering) standing water*												
Removed standing water in past week	90.3 (531)	90.5	87.2		91.8	85.5		93.9	85.6		91.3	90.2
Has not in past week; reports action generally	1.2 (7)	1.1	2.6		0.9	2.2		1.3	1.2		1.9	0.8
Has not in past week; does report action generally	8.5 (50)	8.4	10.3		7.3	12.3		4.7	13.2		6.8	9.0
Total no.	588	546	39		438	138		297	243		377	206
p value		0.516			**0.032**			**0.001**			0.637	
Spraying home (or yard) for mosquitoes												
Sprayed for mosquitoes (self or service)	43.1 (569)	43.7	33.7		44.4	37.7		42.6	43.2		82.3	22.9
No home spraying	56.9 (752)	56.3	66.3		55.6	62.3		57.4	56.8		17.7	77.1
Total no.	1,321	1,222	95		971	321		615	595		446	873
p value		0.058			**0.036**			0.835			**<0.001**	
Larvicide application†												
Has applied larvicide around home (self or family)	31.3 (308)	24.2	10.8		40.5	7.9		30.0	32.9		20.1	37.3
Never applied larvicide around home (self or family)	68.7 (675)	75.8	89.2		59.5	92.1		70.0	67.1		79.9	62.7
Total no.	983	1,229	93		708	253		476	423		334	641
p value		**0.002**			**<0.001**			0.364			**<0.001**	
Installing window or door screens												
Reports putting screens on windows, doors	17.8 (236)	17.4	22.1		17.6	18.5		18.0	18.7		18.1	17.5
Does not report putting screens on windows, doors	82.2 (1,093)	82.6	77.9		82.4	81.5		82.0	81.3		81.9	82.5
Total no.	1,329	1,230	95		976	324		616	600		448	873
p value		0.247			0.715			0.771			0.803	

*All data indicate percentages unless otherwise indicated. Statistically significant differences (p<0.05 by Mann-Whitney U nonparametric test) are shown in boldface. WIC, Women, Infants, and Children Program (US Department of Agriculture Food and Nutrition Service); ZPK, Zika prevention kit. †Among those having yards for which they are responsible, and where water was present.

**Figure 2:**
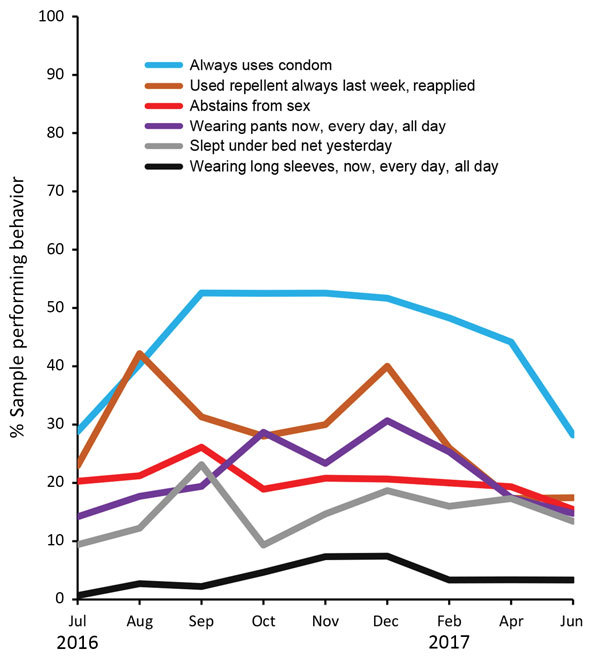
Percentage of women reporting highest levels of 6 Zika personal protection behaviors, by interview month, Puerto Rico, July 2016–June 2017. August 12, 2016: President declares Zika in Puerto Rico a “public health emergency” (https://www.reuters.com/article/us-health-zika-usa/u-s-declares-a-zika-public-health-emergency-in-puerto-rico-idUSKCN10N2KA). September 30, 2016: Free residential spraying discontinued. Women who report the offer through December are referring to receiving the offer before September. October 28, 2016: First baby born with microcephaly in Puerto Rico (https://www.nytimes.com/2016/10/29/health/zika-microcephaly-puerto-rico.html). June 5, 2017: Zika epidemic declared over by Puerto Rico Department of Health (https://www.businesswire.com/news/home/20170605006235/en/Puerto-Rico-Department-Health-Declared-2016-Zika). WIC, Women, Infants, and Children Program (US Department of Agriculture Food and Nutrition Service).

Over the monthly interview cohorts, the top level of condom use rose steadily with a sustained peak at over 50%, whereas mosquito repellent use rose to 42%, declined, and peaked again in December (Figure 2). Wearing long pants had 2 peaks (in October and December) near 30%, then a steep decline in 2017, whereas sexual abstinence stayed near 20%. Bed net use peaked at 23% in September, then fluctuated.

### Zika Home Protection Behaviors

**Figure 3:**
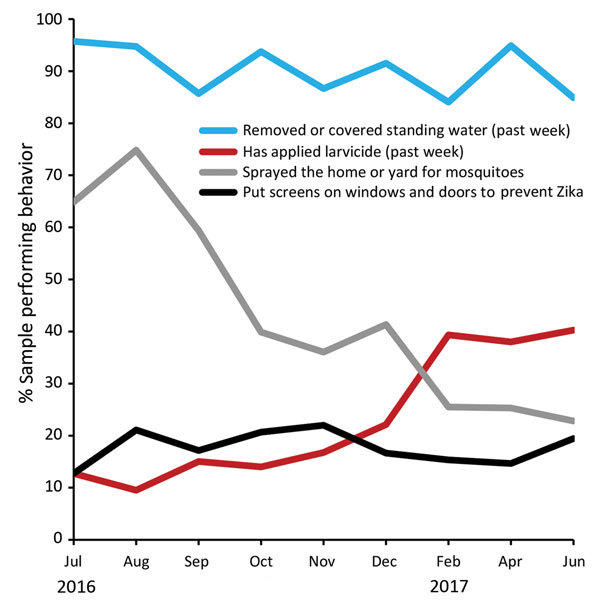
Percentage of women reporting highest levels of 4 Zika home protection behaviors, by interview month, Puerto Rico, July 2016–June 2017. August 12, 2016: President declares Zika in Puerto Rico a “public health emergency” (https://www.reuters.com/article/us-health-zika-usa/u-s-declares-a-zika-public-health-emergency-in-puerto-rico-idUSKCN10N2KA). September 30, 2016: Free residential spraying discontinued. Women who report the offer through December are referring to receiving the offer before September. October 28, 2016: First baby born with microcephaly in Puerto Rico (https://www.nytimes.com/2016/10/29/health/zika-microcephaly-puerto-rico.html). June 5, 2017: Zika epidemic declared over by Puerto Rico Department of Health (https://www.businesswire.com/news/home/20170605006235/en/Puerto-Rico-Department-Health-Declared-2016-Zika). WIC, Women, Infants, and Children Program (US Department of Agriculture Food and Nutrition Service).

We ranked home protection behaviors, from the most frequent (removing standing water [90%]) to the least (installing window or door screens [18%]) (Table 5). Over time, removing standing water declined slightly through September but then remained at >85%, whereas spraying the home for mosquitoes had a steep decline during August–June 2017 (Figure 3). In contrast, larvicide application began low (13%) and then increased through June 2017 (40%).

### Independent Associations between Interventions and Zika Prevention Behaviors

In multivariable logistic regression models, we observed a strong association between the offer of free residential mosquito spraying services and spraying the home for mosquitoes (Table 6). We also observed strong associations between ZPK receipt and larvicide application and between ZPK receipt and bed net use.

**Table 6:** Logistic regression models for Zika prevention behaviors performed by pregnant women that were significantly associated with >1 Zika prevention interventions, Puerto Rico, July 2016–June 2017*†

Behavior	Odds ratio (95% CI)			
	Received ZPK	Recalled Detén el Zika campaign	Received WIC Zika orientation	Offered free residential spraying
Personal protection behaviors				
Bed net use	**3.1 (1.9–5.1)**	1.2 (0.8–1.7)	2.2 (1.0–4.8)	NA
Condom use‡	1.4 (0.9–2.1)	1.4 (1.0–2.0)	**2.4 (1.2–4.7)**	NA
Mosquito repellent use	**1.5 (1.1–2.0)**	**1.6 (1.2–2.1)**	1.2 (0.8–1.9)	NA
Sexual abstinence	0.9 (0.6–1.4)	0.9 (0.6–1.3)	1.2 (0.5–2.5)	NA
Wearing long sleeves	1.9 (0.6–6.2)	2.9 (0.9–8.8)	1.9 (0.2–14.9)	NA
Wearing long pants	1.1 (0.7–1.7)	1.0 (0.7–1.5)	1.4 (0.6–3.0)	NA
Home protection behaviors				
Larvicide application	**8.0 (4.8–13.3)**	0.8 (0.6–1.1)	**2.7 (1.4–5.5)**	**0.4 (0.3–0.5)**
Spraying home or yard for mosquitoes	**1.5 (1.1–2.3)**	1.0 (0.7–1.4)	1.6 (0.9–2.9)	**13.1 (8.5–20.3)**
Removing or covering standing water	2.2 (0.8–5.7)	**2.7 (1.1–6.5)**	0.5 (0.1–4.4)	1.1 (0.4–2.9)
Installing window or door screens	0.8 (0.6–1.2)	0.8 (0.6,1.2)	0.7 (0.4–1.5)	0.9 (0.6–1.5)

*Bold indicates significant result. WIC, Women, Infants, and Children Program (US Department of Agriculture Food and Nutrition Service); ZPK, Zika prevention kit. †Models for WIC orientation and offer of free residential spraying were modeled separately, whereas ZPK distribution and Detén el Zika recall were modeled together to measure independent effects. Thus, each Zika prevention behavior had 3 models. To reduce possible bias associated with sparse data, calendar month of interview was consolidated into a 3-level, 3-month variable. All 5 demographic variables and consolidated calendar month of interview were controlled for in each model, except for the following cases: 1) WIC orientation did not include any calendar month of interview variable; or 2) very few respondents did not receive WIC orientation, thus the naturally occurring control group was very small. To not bias the models, no time of interview variable was included in models of WIC orientation. Education was excluded from bed net, larvicide, and repellent use models. Because of the substantial amount of missing data for education, additional testing was performed to determine whether women with missing education data performed the 10 behaviors with significantly higher or lower frequency. Three behaviors (repellent, bed net, and larvicide use) were significantly associated with whether education data were missing, so education was not included in these models. No calendar month or consolidated month variable was used for any of the larvicide use models because of small cell sizes. ‡Among those reporting having had sex during pregnancy.

## Discussion

For each intervention, exposure patterns corresponded with implementation history; WIC orientation exposure was consistently high, Detén el Zika campaign exposure grew over time, ZPK exposure faltered (because of logistical problems with kit distribution) and then recovered, and the free offer of home mosquito spraying was widely received during the offer period. These largely successful implementations illustrate the benefits of collaborating with a trusted local partner like WIC. WIC was able to incorporate Zika orientations into its regular programming, distribute ZPKs effectively, and provide the free offer of home spraying a WIC visit. WIC also played an important role in developing the Detén el Zika messaging.

Performance of Zika prevention behaviors varied widely. Nearly all women removed any standing water that they saw, and about three quarters usually or always used mosquito repellent, but very few wore long sleeves or put up screens. These findings are consistent with the Pregnancy Risk Assessment Monitoring System Zika Postpartum Emergency Response (PRAMS-ZPER) study of postpartum women in Puerto Rico (*22*). Despite important methodologic differences between PRAMS-ZPER and our analysis, reported frequencies were similar for mosquito repellent use, removing standing water, bed net use, and wearing long sleeves. Where frequencies diverged (condom use and spraying home for mosquitoes), WIC sample frequencies were more similar to PRAMS-ZPER when limited to women in their third trimester. In contrast, interview data from US Virgin Islands in late 2016 (*7*) showed lower frequencies of using repellent, using condoms, removing standing water, and spraying home for mosquitoes. Only data for bed net use were similar to the results of our analysis.

Overall, the ZPK distribution had the greatest number of independent positive associations with Zika prevention behavior and some of the strongest associations. This finding is consistent with a small but growing body of literature demonstrating the effectiveness of distributing items for encouraging prevention behavior (*11*,*23*,*24*). Prevention kits containing prevention products for at-risk populations should be considered a best practice, particularly in low-resource settings.

Larvicide use and bed net use were independently associated with ZPK receipt, and distributing items associated with these 2 largely unfamiliar behaviors probably increased use because women were then able to try them. According to Rogers’ diffusion of innovations theory (*25*), the ability to try a new behavior and observe the results enhances the likelihood of adoption. Larvicide application might have been further enhanced by what Rogers calls “relative advantage”; that is, the intervention might have been popular because it was easier to implement than the other 3 recommended home protection behaviors (removing standing water, installing screens, and spraying home for mosquitoes). Many of the ZPKs in the early months of tracking were missing larvicide tablets; thus, the dramatic increase in larvicide use over the period is not surprising. The finding also suggests that the actual association between ZPKs and larvicide use is stronger than what our results indicate, given that the incomplete kits might have diluted the observed association.

Offer of free residential mosquito spraying services was strongly associated with spraying the home for mosquitoes, enabling women to overcome both cost and logistical barriers. Although efficacy concerns led to discontinuation of the spraying program, the offer had a strong association with spraying behavior, a finding consistent high percentage (81%) of respondents who rated the offer of insecticide spraying to pregnant women as very important.

The Detén el Zika campaign had the greatest independent effect on removing standing water, significant effects for repellent use, and modest (marginally significant) effects for condom use, whereas the WIC orientation appeared to have a slightly greater effect on condom use. Although WIC Zika orientation did not yield the same large number of positive associations in regression models as was observed in the bivariate analyses, its highly successful implementation left it with a very small natural control group, which might have limited the utility of modeling for this intervention.

As we consider the public health implications of these results, we should note that in the context of cross-sectional data with outcomes that are not rare, ORs do not equate to relative risk. Thus, we cannot say that women receiving the free offer of home mosquito spraying were 13 times more likely to spray their homes. Unfortunately, estimating relative risks from ORs is not straightforward. Simple conversion formulas (*26*) have been shown to be imprecise (*27*), but such conversions can provide at least a rough sense of the extent to which relative risk is more modest than odds with nonrare outcomes (*28*). For example, the ORs of 8.0 (ZPK exposure and larvicide application), 13.1 (offer of free residential spraying and spraying home for mosquitoes), and 3.1 (ZPK exposure and bed net use) roughly convert to risk ratios of 5.2, 3.5, and 2.7, respectively, whereas the more modest ORs of 2.7 (Detén el Zika campaign exposure and removing standing water and WIC orientation and larvicide application) and 2.4 (WIC orientation and condom use) undergo a smaller adjustment (1.1, 2.2 and 1.7, respectively). Further research is needed to evaluate these associations more precisely.

In our exploration of intervention mechanisms, the 2 interpersonal communication variables showed stronger association with the interventions and to the Zika prevention behaviors than did the individual variables (Zika concern, perceived likelihood of infection, and self-confidence). This finding suggests that the interpersonal factors were more influential on behavior than individual risk perceptions. Interpersonal communication has long been recognized as an important mediator of the effects of educational campaigns on health-related behavior change (*29**–**33*), and our results confirm this assertion.

The main challenge of this analysis was that the data were collected during an emergency response for nonresearch purposes, meaning that much of the analysis design had to be created after the fact, particularly the creation of Zika behavior outcome variables. Further, this analysis did not use an optimal research design (i.e., there were no pre–post groups or predesignated control groups). The resulting imbalances in naturally occurring control groups prevented the use of a single logistic model for all 4 interventions. However, the use of random sampling from a frame representing 87% of the island’s pregnant women and logistic regression modeling to control confounding by demographic factors provide a credible first look at possible effects of Zika prevention interventions during an epidemic response.

Among the 4 intervention strategies, ZPK distribution appears to have significant independent effects on the greatest number of Zika prevention behaviors. Consistent with the literature, this intervention should be considered a best practice for behavioral support in infectious disease outbreaks, particularly in low-resource settings. Social context factors appeared to be more influential in Zika prevention behavior than personal risk assessment and self-efficacy factors, whereas Zika prevention behaviors that enable women to try out lesser-known behaviors appeared to garner greater acceptance than other behaviors. Areas for future research include developing the evidence base for Zika prevention behavior effectiveness and more precise quantification of intervention mechanisms and effects.

Technical Appendix Supplemental information on development of Zika prevention behavior scales and logistic regression models.
