# Supplementary material for: Influences of Community Interventions on Zika Prevention Behaviors of Pregnant Women, Puerto Rico, July 2016–June 2017
Source: Emerg Infect Dis. 2018 Dec;24(12):2251–61. doi: 10.3201/eid2412.181056 (PMC6256384; doi:10.3201/eid2412.181056)
Supplement: Technical Appendix — Supplemental information on development of Zika prevention behavior scales and logistic regression models. [file 18-1056-Techapp-s1.pdf]

Article DOI: https://doi.org/10.3201/eid2412.181056

Influences of Community Interventions on Zika Prevention Behaviors of Pregnant Women, Puerto Rico, July 2016–June 2017

Technical Appendix

Technical Appendix Table 1. Source questions for Zika prevention behavior scales, personal protection

Base question	Question(s) added to increase scale levels	Final behavior scale
Bed net use: "Yesterday, did you sleep under a mosquito bed net every time you were asleep or took a nap?" Responses:<br>1.No<br>2.Yes	"What actions have you taken to protect yourself from being infected with the Zika virus?" Responses: Used mosquito net (night or day)<br>1. No (didn't choose this response)<br>2. Yes	1. Didn't use yesterday, doesn't report bed net use as prevention step<br>2. Didn't use yesterday, reports bed net<br>3. use as prevention step<br>4. Slept under bed net yesterday
Condom use (among women reporting sexual activity):"When you had sex, how often did you use a condom?"<br>Responses:<br>1.Never<br>2.Sometimes<br>3.Always	None	1. Never<br>2. Sometimes<br>3. Always
Mosquito repellent use: "How often did you use mosquito repellent in the past week?" Responses:<br>1. Never<br>2. Rarely/seldom<br>3. Usually/most of the time<br>4. Sometimes<br>5. Always	A "reapplied repellent: Yes/No" variable was created using the question, "What time or times of the day did you apply repellent?" (Responses: Morning, middle of the day, early afternoon, afternoon, early evening, evening, AM Mid-day and PM, before/when I go outside, after a bath or shower, multiple times per day.) Combinations of responses were used to calculate a value for reapplied repellent,<br>1. No<br>2.Yes	1. Never<br>2. Rarely/seldom<br>3. Sometimes<br>4. Usually/most of the time<br>5. Always, did not report reapplying<br>6. Always and reported reapplying repellent
Sexual abstinence: "Since you became pregnant, have you had sexual intercourse, that is, vaginal, anal, or oral sex, with any male partner?"<br>1. No<br>2. Yes	None	1. Had no sexual relations during pregnancy<br>2.Had sexual relations during pregnancy
Wearing long pants: "Are you wearing long pants right now?" Responses: If yes, "Do you wear long pants every day?"<br>Responses:<br>1. No,<br>2. Yes-part of the day<br>3. Yes-all day	None	1. Not wearing long pants now<br>2. Wearing now, does not wear every day<br>3. Wearing now, wears every day, part of day<br>4. Wearing now, wears every day, all day
Wearing long sleeved shirt: "Are you wearing a long-sleeved shirt right now? If yes, "Do you wear long sleeves every day? Responses:<br>1. No,<br>2. Yes-part of the day<br>3. Yes-all day	None	1. Not wearing long sleeves now<br>2. Wearing now, does not wear every day<br>3. Wearing now, wears every day, part of day<br>4. Wearing now, wears every day, all day

Technical Appendix Table 2. Source questions for Zika prevention behavior scales, home protection

Base question	Question(s) added to increase scale levels	Final behavior scale
Installing window or door screens: "What actions have you taken to protect yourself from being infected with the Zika virus? Responses: Put screens on windows and doors<br>1. No (didn't choose this response)<br>2. Yes	None	1. Reports not putting screens on windows and doors<br>2. Reports putting screens on windows and doors
Larvicide application: "Have you ever put a mosquito dunk in accumulated water around your home?" Responses:<br>1. No<br>2. Yes (Women reporting no yard or no standing water were removed from this scale entirely.)	None	1. Never applied larvicide around home (self/family)<br>2. Has applied larvicide around home (self/family)
Removing (or covering) standing water: "In the past week, have you or somebody in your household removed accumulated water and covered up or screened water containers inside and around your home (on your property)?" Responses:<br>1. No<br>2. Yes (Women who reporting no yard or no standing water were removed from this scale entirely.)	"What actions have you taken to protect yourself from being infected with the Zika virus? Responses: Put cover(s) over the water source/storage unit/water container(s)<br>1. No (didn't choose this response)<br>2. Yes	1. Has not in past week; doesn't report removing standing water as prevention action (in general)<br>2. Has not in past week; reports removing standing water as prevention action<br>3. Removed or covered standing water in past week
Spraying home (or yard) for mosquitoes: Have you received spraying services at your home? Responses:<br>1. No<br>2. Yes	a. "What actions have you taken to protect yourself from being infected with the Zika virus? Responses: sprayed or fumigated (inside or outside) my home<br>1. No (didn't choose this response)<br>2. Yes<br>b. "When services were offered to you, did you accept them?" (for women who are in process, or who arranged for them, but the sprayers haven't arrived).<br>Responses:<br>1. No<br>2. Yes<br>c. "Is there anything that we haven't discussed that you have been doing to reduce the risk?" Response: I have sprayed my house with pesticide by myself or my family<br>1. No<br>2. Yes	1. Has not sprayed for mosquitoes (self or commercial services)<br>2. Sprayed for mosquitoes (self or commercial services)

Technical Appendix Table 3. Personal and family/ social characteristics screened for possible inclusion in logistic models for Zika personal protection behaviors, July 2016–June 2017, Puerto Rico*

	p value					
Personal and risk assessment characteristics†	Mosquito repellent use	Condom use	Wearing long pants	Wearing long sleeve shirt	Sexual abstinence	Bed net use
Having had at least one Zika test‡	0.688	**0.003**	**0.013**	**0.019**	0.058	0.672
Perceived likelihood of Zika virus infection during pregnancy	0.180	0.715	0.817	**0.013**	0.293	**0.049**
Confidence in ability to protect self, baby from Zika infection	**<0.001**	**0.023**	0.397	0.806	0.103	0.278
Level of reported concern about Zika	**<0.001**	0.055	0.615	0.088	0.354	0.630
Family and social environment characteristics§						
Frequency of discussing Zika with friends or family¶	**<0.001**	**<0.001**	**<0.001**	**0.015**	**0.000**	0.988
Aware of Zika prevention activities among family members	**<0.001**	**0.022**	**<0.001**	0.072	0.923	0.746
Aware of Zika prevention activities within neighborhood	**0.005**	**0.031**	**0.004**	**0.018**	0.793	0.277
Aware of Zika prevention activities within municipality	0.174	0.105	0.170	0.730	**0.042**	0.517
Women recall Zika prevention messages on TV news	0.442	**0.036**	0.495	0.617	0.889	0.285
Aware of Zika prevention activities by PR Dept. Health	0.554	0.315	0.143	0.777	**0.048**	0.155
Women recall Zika prevention messages from their doctor	0.525	0.054	0.328	0.251	0.178	0.690
Close friend or relative who is Zika positive	0.070	0.992	0.236	0.556	0.945	0.942
Close friend or relative who is Zika positive and pregnant	0.091	0.668	0.845	0.970	0.141	0.597

*Associations between characteristic-behavior pairs were tested using either Kruskall-Wallis or Mann Whitney U nonparametric tests. Statistically significant associations (p<.05), are in boldface. Characteristics that were statistically significant for association with these ordinal scales were then tested individually with the corresponding dichotomous Zika prevention behavior variable for inclusion in logistic regression models.
†Except where otherwise indicated, all personal and risk assessment characteristics have a five-item response scale ("not at all…" to "extremely…").
‡Two-item "yes-no" response scale.
§Except where otherwise indicated, all family and social environment characteristics have a two-level, "yes-no" response scale.
¶Five-item response scale ("not at all…" to "extremely…").

Technical Appendix Table 4. Personal and family/ social characteristics screened for possible inclusion in logistic models for Zika home protection behaviors,* July 2016–June 2017, Puerto Rico

	p value			
Characteristic	Home mosquito spraying	Removing/ covering standing water	Applying larvicide	Putting screens on doors, windows
Personal and risk assessment characteristics†				
Having had at least one Zika test‡	**0.011**	0.123	**0.001**	0.860
Perceived likelihood of Zika virus infection during pregnancy	**0.001**	0.140	0.131	0.842
Confidence in ability to protect self, baby from Zika infection	0.734	0.726	**0.004**	0.892
Level of reported concern about Zika	0.088	0.098	0.117	0.516
Family and social environment characteristics§				
Frequency of discussing Zika with friends or family¶	**0.002**	**0.004**	0.082	0.136
Aware of Zika prevention activities among family members	**0.006**	**0.014**	0.449	0.090
Aware of Zika prevention activities within neighborhood	0.094	**0.088**	0.890	0.076
Aware of Zika prevention activities within municipality	0.344	**0.841**	0.668	0.921
Women recall Zika prevention messages on TV news	**0.003**	**0.213**	0.242	0.213
Aware of Zika prevention activities by PR Dept. Health	0.319	**0.042**	0.244	0.099
Women recall Zika prevention messages from their doctor	0.212	0.138	0.193	0.214
Close friend or relative who is Zika positive	0.096	0.075	0.447	0.108
Close friend or relative who is Zika positive and pregnant	0.105	0.082	0.610	0.736

*Associations between characteristic-behavior pairs were tested using either Kruskall-Wallis or Mann Whitney U nonparametric tests. Statistically significant associations (p<.05), are in boldface. Characteristics that were statistically significant for association with these ordinal scales were then tested individually with the corresponding dichotomous Zika prevention behavior variable for inclusion in logistic regression models.
†Except where otherwise indicated, all personal and risk assessment characteristics have a five-item response scale ("not at all…" to "extremely…").
‡Two-item "yes-no" response scale.
§Except where otherwise indicated, all family and social environment characteristics have a two-level, "yes-no" response scale.
¶Five-item response scale ("not at all…" to "extremely…").
